# Supplementary material for: Meeting materials from the 2003 Annual Meeting of the International Society for the Prevention of Tobacco Induced Diseases
Source: Tob Induc Dis. 2003 Dec 15;1(4):234. doi: 10.1186/1617-9625-1-4-234 (PMC2671532; doi:10.1186/1617-9625-1-4-234)
Supplement: Additional file 1 [file 1617-9625-1-4-234-S1.zip › Abstract 28-Prevention and Cessation Interventions for Youth.pdf]

Abstract 28

Prevention and Cessation Interventions for Youth

Steve Sussman*, University of Southern California, USA

This presentation will provide a summary of the effectiveness of school-based, family-based, mass media, regulatory, and community efforts on the prevention and cessation of tobacco use among teens.

Based on this summary, needs for future research will be discussed. An attempt will be to try to impart a general picture of the applications of tobacco programming on youth.
