# Supplementary material for: Meeting materials from the 2003 Annual Meeting of the International Society for the Prevention of Tobacco Induced Diseases
Source: Tob Induc Dis. 2003 Dec 15;1(4):234. doi: 10.1186/1617-9625-1-4-234 (PMC2671532; doi:10.1186/1617-9625-1-4-234)
Supplement: Additional file 1 [file 1617-9625-1-4-234-S1.zip › Abstract 29-The Impacts of Smoking Bans on Cessation and Relapse.pdf]

Abstract 29

The Impacts of Smoking Bans on Cessation and Relapse

Daniel R Longo*, University of Missouri-Columbia, USA

Concerns with second hand smoke have been the major force in the promotion and adoption of workplace smoking bans.

However, a number of studies report a positive side benefit of these bans --- smokers quit.

Using the framework developed in the author's own work on this topic, this session reviews the trends reported in the literature as well as in the lay media.

This history of smoking bans as reflected in the literature highlights the various models that have been implemented, including their effectiveness.

Factors that facilitate these bans are also identified and discussed.
