# Supplementary material for: Meeting materials from the 2003 Annual Meeting of the International Society for the Prevention of Tobacco Induced Diseases
Source: Tob Induc Dis. 2003 Dec 15;1(4):234. doi: 10.1186/1617-9625-1-4-234 (PMC2671532; doi:10.1186/1617-9625-1-4-234)
Supplement: Additional file 1 [file 1617-9625-1-4-234-S1.zip › Abstract 31-Smoking and Smoking Cessation in Pregnancy.pdf]

Abstract 31

Smoking and Smoking Cessation in Pregnancy - Analysis of Safety and Efficacy

Gideon Koren*, University of Toronto, Canada

Smoking in pregnancy is a major public health issue, adversely affecting the health of millions of children worldwide.

While many women are motivated to quit smoking in pregnancy, many cannot do so due to the addictive nature of nicotine dependence.

Nicotine replacement therapy has not proven effective in pregnancy, possibly due to the low dose used and the increase in nicotine metabolism in late pregnancy.

We have recently shown the apparent effectiveness of buproprion in pregnancy.

Coupled with increased evidence of its' safety, this drug may become an important component in the battle against fetal exposure to smoking.
