# Supplementary material for: Meeting materials from the 2003 Annual Meeting of the International Society for the Prevention of Tobacco Induced Diseases
Source: Tob Induc Dis. 2003 Dec 15;1(4):234. doi: 10.1186/1617-9625-1-4-234 (PMC2671532; doi:10.1186/1617-9625-1-4-234)
Supplement: Additional file 1 [file 1617-9625-1-4-234-S1.zip › Abstract 32-Genotoxic Effects of Smoking on Human Gametes and Embryos.pdf]

Abstract 32

Genotoxic Effects of Smoking on Human Gametes and Embryos

Maria Teresa Zenzes*, Department of Obstetrics and Gynaecology, Division of Reproductive Sciences, University of Toronto, Toronto, Canada

Tobacco smoking adversely affects reproductive function. Epidemiological studies have shown consistent, dose-related associations of smoking with delay of conception, increased risk of abortion, and early age of menopause. Recent investigations have been aimed at understanding these deleterious effects of smoking. Reproductive technologies have been useful for providing clinical data on reproductive outcome and biological material such as reproductive fluids, spare oocytes, spermatozoa, and spare early embryos.

Components of cigarette smoke, such as cadmium (a heavy metal), nicotine (a toxic alkaloid) and its metabolite cotinine, were detected in dose-relationship with smoking in ovarian granulosa cells, and in follicular fluids and seminal plasma. This gonadal environment in smokers has been shown to be detrimental to the development and viability of their gametes; furthermore, it induces genetic damage. Heavy female and male smoking is associated in dose-relationship with ***i)*** reduced numbers of retrieved oocytes (by 8% to 17%); ***ii)*** reduced density of ejaculated spermatozoa (by 13% to 18%); ***iii)*** alterations in the meiotic spindle leading to chromosomal errors in oocytes (diploidy) and in spermatozoa (aneuploidy). In oocytes of mice exposed during meiotic maturation *in vitro* to nicotine or cadmium, severe alterations of meiotic spindle structure and chromosome alignment were also found.

Benzo(a)pyrene [B(a)P], a carcinogenic polycyclic aromatic hydrocarbon, is produced from cigarette combustion. Its reactive metabolite binds covalently to DNA, forming adducts. B(a)P-DNA adducts were detected, in elevated levels, in ovarian granulosa cells of smoker women, in spermatozoa of smoker males, and in preimplantation embryos of smoking couples. Transmission of DNA-adducts to embryos was found to be mainly by sperm. This may be related to many divisions occurring in spermatic cells and/or to a low capacity of mature spermatozoa to repair prezygotic DNA damage.
