# Supplementary material for: Meeting materials from the 2003 Annual Meeting of the International Society for the Prevention of Tobacco Induced Diseases
Source: Tob Induc Dis. 2003 Dec 15;1(4):234. doi: 10.1186/1617-9625-1-4-234 (PMC2671532; doi:10.1186/1617-9625-1-4-234)
Supplement: Additional file 1 [file 1617-9625-1-4-234-S1.zip › Abstract 33-The Effects of Tobacco Smoking on Fetal Lung Surfactant and Pulmonary.pdf]

Abstract 33

The Effects of Tobacco Smoking on Fetal Lung Surfactant and Pulmonary Development

J. Elliott Scott*, Departments of Oral Biology & Anatomy, Faculties of Medicine & Dentistry, University of Manitoba and the Biology of Breathing Group, Lung Development Section, Manitoba Institute of Child Health, Winnipeg, Canada

Smoke from tobacco, marijuana or other sources has dramatic effects on lung. While it is generally accepted that smoke exposure, particularly from cigarette tobacco is the major cause of lung cancer, recent research is beginning to establish much more subtle effects on lung and lung cells. As the lung is the chief route of body exposure to the environment and presents only an extremely attenuated barrier to foreign agents or toxic materials such as fungal spores or smoke, it is perhaps not surprising that pulmonary smoke inhalation has the potential to alter the milieu of the air-exchange tissues and passages. What is surprising is the lack of information about the mechanisms by which smoke exerts these effects. The lung is lined by a phospholipid-rich material, the pulmonary surfactant that forms the first line of defense against inhaled foreign materials. For many years surfactant was thought to be a passive participant in lung function. New research indicates that pulmonary surfactant has many functions, and undergoes rapid and continuous changes in a cycle of synthesis, secretion and reutilization. Furthermore the surfactant is a vital part of the complex lung environment, not only at the level of the air exchange tissues but far up the bronchial tree where it interacts with the mucus layer. At birth lung surfactant acts to enable lung expansion at the first breath, to stabilize the alveoli such that they do not collapse and to reduce surface tension. Any insult that alters this functional relationship can lead to neonatal Respiratory Distress, the leading cause of mortality among premature infants. Smoke exposure in the adult appears to induce DNA damage in lung cells and reduce important components of the pulmonary surfactant, namely surfactant proteins (SP-A, SP-B and SP-D) and disaturated phosphatidylcholine, both of which are vital for function. In the developing fetus, with some evidence to the contrary, maternal smoking appears to be associated with development of asthma, reduced lung volume and alveolar numbers, changes in surfactant phospholipids and SP-A. In addition components of tobacco smoke interact with α_7-nicotinic acetylcholine receptors on pulmonary neuroendocrine cells, inducing serotonin release which may be associated with development of pediatric pulmonary disease. Overall evidence indicates that maternal smoking is detrimental to lung development and may interfere with appropriate maturation of the pulmonary surfactant, thereby jeopardizing fetal viability at birth. Furthermore maternal smoking may be related to development of pulmonary diseases such as asthma and reduced lung function in childhood.
