# Supplementary material for: Meeting materials from the 2003 Annual Meeting of the International Society for the Prevention of Tobacco Induced Diseases
Source: Tob Induc Dis. 2003 Dec 15;1(4):234. doi: 10.1186/1617-9625-1-4-234 (PMC2671532; doi:10.1186/1617-9625-1-4-234)
Supplement: Additional file 1 [file 1617-9625-1-4-234-S1.zip › Abstract 34-Smoking Relapse One Year After Delivery Among the Women Participating in.pdf]

Abstract 34

Smoking Relapse One Year After Delivery Among the Women Participating in Smoking Cessation Intervention During Pregnancy

Wojtek Hanke* and Kinga Polanska, Nofer Institute, Lodz, Poland

Introduction: The exposure to environmental tobacco smoke gives rise to an excessive risk of several diseases in infancy and childhood and may increase the risk of Sudden Infant Death Syndrome, respiratory system diseases, allergy and asthma. It is encouraging that approximately 30% of women who continue smoking after getting pregnant, tend to quit smoking during pregnancy. Unfortunately, 80-90% will return to smoking by 12 months postpartum.

Aim: The aim of this study was to evaluate smoking relapse after delivery in women quitting smoking in pregnancy.

Methods: The study was performed between 1 December, 2001 and 31 December, 2002, as stage II of the smoking cessation intervention for pregnant women from the Lodz region (central Poland). This time we collected data on smoking relapse among women who were covered by midwife-assisted anti-smoking intervention during pregnancy and compared them with the findings for controls who received standard written information on health hazard from smoking. The midwives who previously conducted the intervention interviewed the women from the intervention and control group about their smoking status to find whether any smoking relapse had occurred within a one-year period since the delivery.

Results: At stage I of the project, the proportion of women who quitted smoking in pregnancy was 34.2% in the intervention group and 15.9% in the controls. One year postpartum, the percentage of smoking women amounted to 64.8 in the intervention group and 79.9 in the controls ($p=0.002$). No statistically significant differences were noted between the two groups with respect to smoking relapse 12 months after delivery ($p=0.2$).

Conclusions: The findings indicate that even an intensive antismoking counselling during pregnancy does not protect women from returning to smoking postpartum.
