# Supplementary material for: Meeting materials from the 2003 Annual Meeting of the International Society for the Prevention of Tobacco Induced Diseases
Source: Tob Induc Dis. 2003 Dec 15;1(4):234. doi: 10.1186/1617-9625-1-4-234 (PMC2671532; doi:10.1186/1617-9625-1-4-234)
Supplement: Additional file 1 [file 1617-9625-1-4-234-S1.zip › Abstract 35-Near Infrared Spectroscopy is a Potential Analytical Tool by Which to Measure.pdf]

Abstract 35

Near Infrared Spectroscopy is a Potential Analytical Tool by Which to Measure the Effects of Tobacco Smoke On Periodontal Inflammation.

A.Hynes[1]*, K-Z Liu[2], DL Singer[1], A. Man[2] and DA Scott[1]. University of Manitoba and National Research Council, Canada.

BACKGROUND: Periodontitis is an endemic, plaque-associated chronic inflammatory disease that degrades the tissues surrounding the teeth. Tobacco smokers are particularly susceptible to periodontitis and respond less favorably to periodontal therapy. The electronic transitions stemming from the heme ring and central metal ion of hemoglobin, and water absorptions, are particularly strong in the near infrared (NIR) region.

AIMS: Tobacco smoking is known to influence multiple components of the inflammatory response and information on key inflammatory indices can be extracted from NIR spectra. Therefore, we aimed to establish if NIR spectroscopy could be used to measure major inflammatory indices in the periodontium: *(a)* Periodontal tissue temperature; *(b)* Tissue edema; *(c)* Tissue oxygenation; (d) tissue perfusion.

METHODS: We employed a custom-designed intraoral (*in vivo*) NIR spectroscopy probe to obtain NIR spectra from an inflamed periodontal site and a contralateral healthy periodontal site in 11 subjects.

RESULTS: We were able to identify near infrared spectral shifts indicative of alterations to several inflammatory indices in inflamed periodontal tissues. These were *superficial blood volume* (perfusion), the *tissue hydration index* (edema), and the relative concentrations of *oxygenated hemoglobin* and *deoxygenated hemoglobin* (tissue oxygenation).

CONCLUSIONS: NIR spectroscopy represents a potential in vivo, non-invasive analytical tool by which to measure the effects of tobacco smoke on periodontal inflammation and inflammation at other cutaneous sites.
