# Supplementary material for: Meeting materials from the 2003 Annual Meeting of the International Society for the Prevention of Tobacco Induced Diseases
Source: Tob Induc Dis. 2003 Dec 15;1(4):234. doi: 10.1186/1617-9625-1-4-234 (PMC2671532; doi:10.1186/1617-9625-1-4-234)
Supplement: Additional file 1 [file 1617-9625-1-4-234-S1.zip › Abstract 36-Evaluation of the Kick Butt @ Work Smoking Cessation Program at the.pdf]

Abstract 36

Evaluation of the Kick Butt @ Work Smoking Cessation Program at the Wellness Institute, Manitoba, Canada

Kevin Saunders, Sherry Mooney*, Erica White and Nicole Dunn. The Wellness Institute, Manitoba, Canada

Background: The Wellness Institute @ Seven Oaks General Hospital began offering the successful Kick Butt program in 2000. In 2002 numerous calls from progressive employers looking for cessation supports for staff resulted in further program development. These employers recognized the detrimental effects tobacco use had on people as well as the costs of employing smokers. The Conference Board of Canada has calculated productivity, absenteeism, benefit costs and the cost of maintaining smoking areas to be $2500 per year per smoking employee. Kick Butt @ Work was developed to meet the local need and test the already successful program model in a work place setting.

Program Description: The Kick Butt@ Work program addresses tobacco use as an addiction. The program combines individual and group sessions with fixed intakes, recognizes and treats both the addiction and the behaviour aspects of tobacco use. The program consists of five components: 1) Pharmacology; 2) Behaviour counselling; 3) Replacement activities; and 4) Relapse prevention and aftercare 5) Group support. Program services include a medical assessment, four behaviour therapy sessions and a lifestyle/fitness assessment and evaluation.

Results: In its early stage Kick Butt @ has had a 50% success rate one year post quit. Subsequent groups will require follow up to reveal ongoing quit rates.

Conclusion: Kick Butt @Work has a higher success rate than self-help programs, on-line programs, and other group programs reviewed. It is the most successful program offered to date for employers in the City of Winnipeg.
