# Supplementary material for: Meeting materials from the 2003 Annual Meeting of the International Society for the Prevention of Tobacco Induced Diseases
Source: Tob Induc Dis. 2003 Dec 15;1(4):234. doi: 10.1186/1617-9625-1-4-234 (PMC2671532; doi:10.1186/1617-9625-1-4-234)
Supplement: Additional file 1 [file 1617-9625-1-4-234-S1.zip › Abstract 37-Tobacco Smoking and Periodontal Health in a Maintenance Population.pdf]

Abstract 37

Tobacco Smoking and Periodontal Health in a Maintenance Population.

Kells L*[1], Picard JP[1], Singer DL[1], Gelskey SC[1], Scott DA[1,2] . Dental Diagnostics & Surgical Sciences[1] and Oral Biology[2], University of Manitoba, Winnipeg, Canada.

OBJECTIVES: To examine clinical features of periodontal destruction in smoking and non-smoking adults with periodontitis on long-term maintenance therapy, in a cross-sectional manner. **SUBJECTS AND METHODS:** 73 adult subjects with periodontitis and on long-term maintenance therapy were recruited. The periodontal status of each subject (number of teeth, probing depth [PD], and clinical attachment level [CAL]) was examined, a detailed smoking history was obtained, and expired-air CO levels were measured. **RESULTS:** In current smokers (n = 17), mean overall PD (p = 0.012), interproximal PD (p = 0.018), maxillary PD (p = 0.003) and mean maxillary CAL (p = 0.019) were increased, compared to previous- and never-smokers (n = 56). A trend towards increased overall CAL in smokers (p = 0.067) was also observed. **CONCLUSIONS:** In a population of maintenance therapy patients, periodontitis is more severe in smokers, compared to non-smokers. These results suggest that, in smokers, interproximal and maxillary sites may be particularly susceptible to periodontal destruction. Continuing studies will examine the rate of periodontal disease progression in these same subjects.
