# Supplementary material for: Meeting materials from the 2003 Annual Meeting of the International Society for the Prevention of Tobacco Induced Diseases
Source: Tob Induc Dis. 2003 Dec 15;1(4):234. doi: 10.1186/1617-9625-1-4-234 (PMC2671532; doi:10.1186/1617-9625-1-4-234)
Supplement: Additional file 1 [file 1617-9625-1-4-234-S1.zip › Abstract 38-Tobacco use cessation service practice patterns of Manitoba dentists and.pdf]

Abstract 38

Tobacco use cessation service practice patterns of Manitoba dentists and dental hygienists

Adleman D*[1], Gelskey SC[1] and Brothwell D[2] . Dental Diagnostics & Surgical Sciences[1] and Center for Community Oral Health[2], University of Manitoba, Winnipeg, Canada.

Objectives: Tobacco use is a known risk factor or cause of oral cancer, periodontitis and tooth loss and it compromises oral health treatment. Though tobacco use-cessation counseling (tucc), delivered by oral health professionals, has been shown to significantly increase tobacco use quit rates among patients, many practitioners do not provide this service. Practitioners report 'lack of adequate training' in tucc as a major barrier. The purpose of this research was to determine the extent to which Manitoba dentists and dental hygienists provide tucc services and to identify perceived barriers to providing tucc.

Methods: A questionnaire was sent to all licensed dentists (548) and dental hygienists (569) in Manitoba in June 2003 and a reminder questionnaire to non-respondents (335, 385) in July 2003. Questionnaires collected data regarding tucc practices of oral health practitioners as well as demographic information.

Results: The overall response rate was 52 percent (579/1117), with 54 percent (298/548) for dentists and 49 percent (280/569) for dental hygienists. Preliminary results indicate that compared to earlier graduates, a significantly greater proportion of recent graduates (within the past 4 years) report to have received tucc training. They report to be better prepared to provide tucc services to their patients. In addition, significantly more recent graduates indicate an interest in receiving additional tucc training.

Implications: Results of this research will be used to evaluate the influence of recent tucc training at the Faculty of Dentistry, University of Manitoba as well as to plan programs of continuing education for oral health practitioners.
