# Supplementary material for: Meeting materials from the 2003 Annual Meeting of the International Society for the Prevention of Tobacco Induced Diseases
Source: Tob Induc Dis. 2003 Dec 15;1(4):234. doi: 10.1186/1617-9625-1-4-234 (PMC2671532; doi:10.1186/1617-9625-1-4-234)
Supplement: Additional file 1 [file 1617-9625-1-4-234-S1.zip › Abstract 39-Profiling of Tobacco Use Among Patients Attending a Specialist Dental Clinic.pdf]

Abstract 39

Profiling of Tobacco Use Among Patients Attending a Specialist Dental Clinic.

Picard JP*[1], Borden JT[2], Singer DL[1], Gelskey SC[1], Scott DA[1,2] . Dental Diagnostics &Surgical Sciences[1] and Oral Biology[2], University of Manitoba, Winnipeg, Canada.

Background: Tobacco use data in dental clinics are usually obtained by patient-completed questionnaires, which are known to be unreliable. We hypothesized that tobacco use is under-reported by patients attending a specialty periodontics dental clinic.

Subjects and methods: The smoking status of 108 subjects attending a university-based specialist periodontics clinic was assessed by clinician-administered interview and salivary cotinine measurement.

Results: 10.0% of the detected smokers (salivary cotinine > 14.2 ng ml^{-1}) reported to be non-smokers in the clinician-administered interview, while 23.8% of detected smokers (salivary cotinine > 14.2 ng ml^{-1}) claimed to be non-smokers in the patient-completed questionnaires provided by subjects at the periodontics clinic.

Conclusions: The combination biochemical profiling and clinician-administered tobacco use interview represents an excellent method to improve the detection of an important, periodontally susceptible population – regular smokers who deny smoking.
