# Supplementary material for: Meeting materials from the 2003 Annual Meeting of the International Society for the Prevention of Tobacco Induced Diseases
Source: Tob Induc Dis. 2003 Dec 15;1(4):234. doi: 10.1186/1617-9625-1-4-234 (PMC2671532; doi:10.1186/1617-9625-1-4-234)
Supplement: Additional file 1 [file 1617-9625-1-4-234-S1.zip › Abstract 40-Detection of the effect of nicotine on a pancreatic acinar cell with a.pdf]

Abstract 40

Detection of the effect of nicotine on a pancreatic acinar cell with a photothermal assay

Vladimir P. Zharov [a], Valentive Galitovsky [a] and Parimal Chowdhury [b]
[a]Philips Classic Laser Biomedical Laboratory, University of Arkansas for Medical Sciences (UAMS), Little Rock, AR, 72205
[b] Department of Physiology and Biophysics, UAMS, Little Rock, AR, 72205

Utilization of currently most powerful fluorescent assays is applicable only for targeted cellular structures that are by themselves fluorescent or can be labeled with fluorescent markers. Most potential nicotine targets, however, are nonfluorescent in their native state and pretreatment may lead to unpredictable distortion of cell-nicotine interactions. Our goal in this study was to examine the capability of a new highly sensitive photothermal (PT) assay for rapid detection of the nonspecific influence of nicotine on pancreatic acinar cells (exocrine tumor cell line, AR42J) *in vitro* without fluorescent labelling at the live single-cell level. In this assay, cell response to nicotine was detected through the monitoring of PT signals from light-absorbing, nonfluorescent endogenous cellular structures that can be used as natural indicators for nicotine's action. PT signals are formed by irradiation of a cell with a pump short laser pulse (a tunable parametric pulse laser-OPO, 420-570 nm, 0.1-100 μJ, 8 ns width), and laser-induced local heating of absorbing cellular structures is then recorded with a second collinear probe laser pulse (Raman-Shifter 639 nm, 13 ns width). These signals are sensitive to several parameters of potential indicators, including optical, thermal and geometric properties. The results show that presence of nicotine leads to a change in the structure of PT images of cell as well as photodamage threshold and these effects are dose dependent, the minimum detectable dose is at a nicotine concentration of 1nM. The dependence of PT specific parameters on nicotine concentration was almost linear in the concentration range 1 nM – 100 μM, with saturation maximum at 100 μM-1 mM. Thereafter the PT parameters declined rapidly to control level in the dose range of 1-50 mM. Conventional tests (Trypan blue, Annexin V-PI) performed in parallel has shown no response at concentrations below 1μM and with increase in concentration from 1mM to 50mM, rapid increase of apoptotic and necrotic cells occurred .The mechanism of this dose response of nicotine may be due to 1) high sensitivity of PT response to nicotine –induced changes in light-absorbing, endogenous cellular structures, probably in cell membrane that can be used as natural indicators of nicotine's action and 2) decreased resistance in cell to laser-induced local overheating around such absorbing structures. Supported by grants NSF #BES-0119470, NIH # 1R21 CA 97422-01A1 and Tobacco settlement foundation.
