# Supplementary material for: Meeting materials from the 2003 Annual Meeting of the International Society for the Prevention of Tobacco Induced Diseases
Source: Tob Induc Dis. 2003 Dec 15;1(4):234. doi: 10.1186/1617-9625-1-4-234 (PMC2671532; doi:10.1186/1617-9625-1-4-234)
Supplement: Additional file 1 [file 1617-9625-1-4-234-S1.zip › Abstract 42-Tobacco use by adolescents in Ghana.pdf]

Abstract 42

Tobacco use by adolescents in Ghana.

George Brown-Otoo*, African-American Lung Association, Accra, Ghana.

A study conducted by the African-American Lung Association about the use of tobacco revealed that smoking of cigarettes is the most common form of tobacco used in Ghana, though some people chew or sniff tobacco. The study further revealed data on the prevalence of cigarettes and the use of other forms of tobacco, as well as access/availability.

Findings from the survey showed that 15.3% of students in the junior high school had ever smoked a cigarette. 19.3% usually smoke at home. 46.1% buy cigarettes in a store. 55% who bought cigarettes in a store were not refused purchase because of their age.

Environmental tobacco smoke (ETS):
In relation to environmental tobacco smoke, 12.9% live in homes where others smoke. 39.9% are around others who smoke in places outside their homes. 57.2% think that smoking should be banned from public places. 26.1% have one or more parents who smoke. 6.1% have most or all friends who smoke.

Media and Advertising:
With media and advertising, 50% saw a lot of anti-smoking adverts. Media messages: 40.6% have an object with cigarette brand logos. 15.2% were offered free of charge by tobacco company representatives.

British American Tobacco (BAT) is the leading company operating in Ghana. This industry makes vital use of billboards and hoardings in advertising their products. These billboards and hoardings of various sizes are mounted at vantage points such as main centers, busy roads and highways, entrances to hotels, traffic intersections and crossroads. These billboards, therefore, receive maximum exposure to lure millions of innocent Ghanaian children to die needless deaths.

In 1989 the Government of Ghana made a pronouncement of a ban on smoking in public places. There was a further Directive in 1991, that there should be no smoking on the premises of any Ministry of Health facility throughout Ghana.

To date, there is no legislation on this ban on smoking in public places. This has paved the way for the youth to use this deadly product everywhere in Ghana. This is alarming.
