# Supplementary material for: Meeting materials from the 2003 Annual Meeting of the International Society for the Prevention of Tobacco Induced Diseases
Source: Tob Induc Dis. 2003 Dec 15;1(4):234. doi: 10.1186/1617-9625-1-4-234 (PMC2671532; doi:10.1186/1617-9625-1-4-234)
Supplement: Additional file 1 [file 1617-9625-1-4-234-S1.zip › Abstract 45-The tobacco outreach program at the Tata Institute of Fundamental Research,.pdf]

Abstract 45

The tobacco outreach program at the Tata Institute of Fundamental Research, Mumbai, India.

Pawar Vilas, TIFR, Mumbai, India.

The Tata Institute of Fundamental Research has been engaged in a long-term epidemiological research project in Oral Cancer, pre-cancerous lesions and conditions in the Mumbai region of India.

For this, we selected a 50 000 study population amongst Indian villagers. They were followed every year for their oral check up for any pre-cancerous lesion or cancer & their tobacco habits were also recorded. The dentists from the project took biopsies of pre-cancerous lesions & conditions. The biopsies were examined for histopathological changes were also studied. We used to treat the patients from our study group free of cost for their dental diseases. This strategy was used for prevention of oral cancer as it was observed during our study that one of the reasons for tobacco habit was tooth-pain.

It is considered amongst Indian villagers that tobacco relieves tooth pain. This is the main reason for their tobacco habit. Once the habit is developed its very difficult to give it up due to tobacco addiction.

The link between tobacco use and the development of oral cancers relation is well established.
