# Supplementary material for: Meeting materials from the 2003 Annual Meeting of the International Society for the Prevention of Tobacco Induced Diseases
Source: Tob Induc Dis. 2003 Dec 15;1(4):234. doi: 10.1186/1617-9625-1-4-234 (PMC2671532; doi:10.1186/1617-9625-1-4-234)
Supplement: Additional file 1 [file 1617-9625-1-4-234-S1.zip › Abstract 46-Tobacco Consumption and Exposure of Manitobans (1994 - 2000).pdf]

Abstract 46

Tobacco Consumption and Exposure of Manitobans (1994 - 2000)

Bo Nancy Yu*[1,2], Tom Czyczko[1], and Andrew Loughead[1]
[1] Manitoba Health, [2] Department of Community Health Sciences, Faculty of Medicine, The University of Manitoba, Winnipeg, Manitoba, Canada.

Background: The National Population Health Survey (NPHS) and Canadian Community Health Survey (CCHS) were designed and conducted by Statistics Canada to provide timely information on self-reported health and chronic diseases, health care services needs and utilization, life-style and health determinants. Cigarette smoking and exposure to 2nd hand smoking are common contents of these two surveys.

Objective: To investigate tobacco consumption surveillance at provincial and regional levels for baseline information on descriptive epidemiology of cigarette smoking status and exposure to 2nd-hand cigarette smoke.

Methods: The Manitoba samples from NPHS (1994, 1996 & 1998) and CCHS (2000) were analyzed for the prevalence and time trend of cigarette smoking in Manitoba. CCHS (2000) data were further explored to describe the prevalence of cigarette smoking and exposure to 2nd-hand smoke of Manitobans at the level of health regions. Logistic regression analysis was used to determine the impact of demographic, socio-economic, and life-style risk factors on smoking status.

Results: Approximately 20% of Manitobans were estimated to be daily smokers in 2000, although a wide range of the prevalence of daily smokers (35% to 12%) existed across the health regions. Education, marital status, and income were all significantly related to tobacco consumption and smoking cessation. Since 1994, the proportion of daily smokers in Manitoba appears to have dropped significantly, from 24% to 20%. More smokers are quitting. For example, 41% of male respondents are former smokers in 2000 compared to 29% in 1994. The proportion of people reporting exposure to 2nd-hand cigarette smoke was positively correlated to the prevalence of daily smokers across all health regions. Over 60% of Manitoba residents in the surveys reported that they were bothered or physically irritated by cigarette smoke. Demography, socio-economic status, Life-style and behavioral risk factors were analyzed for their implications in smoking and smoking cessation.
