# Supplementary material for: Meeting materials from the 2003 Annual Meeting of the International Society for the Prevention of Tobacco Induced Diseases
Source: Tob Induc Dis. 2003 Dec 15;1(4):234. doi: 10.1186/1617-9625-1-4-234 (PMC2671532; doi:10.1186/1617-9625-1-4-234)
Supplement: Additional file 1 [file 1617-9625-1-4-234-S1.zip › ISPTID 2003 Conference Organizing.pdf]

Conference 2003

International Society for the Prevention of Tobacco Induced Diseases

Congress President

Dr. David A. Scott, University of Manitoba, Canada

Local Organizing Committee

Dr. Norman Fleming, University of Manitoba, Canada
Dr. Elliott Scott, University of Manitoba, Canada
Yvonne Zarnowski, University of Manitoba, Canada
Dr. Maria Teresa Zenzes, University of Toronto, Canada

Scientific Committee

Dr. Jean-Michel Halimi, Universite de Tours, France
Dr. Teruo Inoue, Dokkyo University, Japan
Dr. Denis Kinane, University of Louisville, USA
Dr. Sarah McGhee, Hong Kong University, China
Dr. Robert Murray, University of Manitoba, Canada
Dr. Steve Sussman, University of Southern California, USA
Dr. Maria T. Zenzes, University of Toronto, Canada
