# Supplementary material for: Meeting materials from the 2003 Annual Meeting of the International Society for the Prevention of Tobacco Induced Diseases
Source: Tob Induc Dis. 2003 Dec 15;1(4):234. doi: 10.1186/1617-9625-1-4-234 (PMC2671532; doi:10.1186/1617-9625-1-4-234)
Supplement: Additional file 1 [file 1617-9625-1-4-234-S1.zip › Abstract 2-Estimates of second-hand smoke exposure based on clinical outcomes and.pdf]

Abstract 2

Estimates of second-hand smoke exposure based on clinical outcomes and biomarkers

Hedley AJ*, McGhee SM, Lam TH
Department of Community Medicine, The University of Hong Kong, Hong Kong

The recent report by Enstrom and Kabat[1] caused further controversy about the health effects of second-hand smoke. However their report does confirm that exposure to secondhand smoke (SHS) causes injury to the respiratory system with the finding of a combined increased mortality risk for men and women for chronic obstructive pulmonary disease (Relative Risk 1.65 [1.0-2.73]. This is consistent with other investigations which demonstrate respiratory system sensitivity to SHS at all ages and in different settings. In Hong Kong, studies have shown that the exposure of infants to SHS *in utero* or postnatally in the home was linked to higher consultation rates and hospitalization for respiratory and other illnesses. Smoking in the home was clearly associated with bronchitic symptoms in a cohort of primary school children, independently of ambient air pollution. In an adult workforce, workplace exposures to passive smoking were associated with significant excess risks (66% to 212%) for all respiratory symptoms and increased health care costs. In a population survey the prevalence of SHS exposures at work was 47.5% among non-smoking full time workers compared with only 26% at home. Those exposed at work were 37% more likely to consult a doctor for respiratory illness. The increased health care costs for primary care alone among three million workers was estimated at US$29M annually. The majority of catering workers in Hong Kong are exposed to second-hand smoke and most have markedly raised urinary cotinine levels, indicating increased life time risks for heart disease and cancer. Four independent case control studies on lung cancer and passive smoking in Hong Kong, reviewed by the United States Environmental Protection Agency, gave an overall relative risk of 1.48 (1.21-1.81).

The evidence that we have epidemics of respiratory disease in Hong Kong caused by secondhand smoke is robust and supported by evidence from several different types of investigation.

Reference

1. Enstrom JE, Kabat GC. Environmental tobacco smoke and tobacco related mortality in a prospective study of Californians, 1960-98. *BMJ* 2003; 326:1057.
