# Supplementary material for: Meeting materials from the 2003 Annual Meeting of the International Society for the Prevention of Tobacco Induced Diseases
Source: Tob Induc Dis. 2003 Dec 15;1(4):234. doi: 10.1186/1617-9625-1-4-234 (PMC2671532; doi:10.1186/1617-9625-1-4-234)
Supplement: Additional file 1 [file 1617-9625-1-4-234-S1.zip › Abstract 3-Infrared Spectroscopy as a Novel Molecular Tool in Quantifying Tobacco.pdf]

Abstract 3

Infrared Spectroscopy as a Novel Molecular Tool in Quantifying Tobacco Smoke Exposure and Identifying Tobacco-Induced Pathologies

Kan-Zhi Liu, National Research Center, Canada

Infrared (IR) spectroscopy can distinguish differences in the characteristics of diverse molecules by using infrared radiation to probe chemical bonds. Consequently, alterations to the molecular characteristics of tissues and body fluids that help define specific pathological processes and conditions can be identified by IR spectroscopy.

Recently, my colleagues and I have used IR spectroscopy (i) to establish that cotinine molecules exhibit a characteristic IR profile, and (ii) to accurately differentiate smokers and non-smokers by identifying tobacco use-associated differences in the IR spectra of serum films.

Major tobacco-induced diseases, in terms of incidence and mortality, are known to include cancers, cardiovascular diseases and periodontitis. Together with my co-workers at the National Research Center of Canada and The Faculty of Dentistry at University of Manitoba, I have recently used IR spectroscopy to diagnose oral squamous cell carcinomas by detecting key alterations to the biochemical profiles of oral tissues; to observe cardiovascular pathological abnormalities; and to generate initial evidence to support the development of IR spectroscopy as a potential diagnostic and prognostic tool for periodontitis.

This presentation, therefore, will comprise an overview of IR spectroscopy as a uniquely multi-dimensional tool by which to investigate various aspects of several tobacco-induced pathologies.
