# Supplementary material for: Meeting materials from the 2003 Annual Meeting of the International Society for the Prevention of Tobacco Induced Diseases
Source: Tob Induc Dis. 2003 Dec 15;1(4):234. doi: 10.1186/1617-9625-1-4-234 (PMC2671532; doi:10.1186/1617-9625-1-4-234)
Supplement: Additional file 1 [file 1617-9625-1-4-234-S1.zip › Abstract 4-Hair Nicotine and Cotinine as Biomarker of ETS.pdf]

Abstract 4

Hair Nicotine and Cotinine as Biomarker of ETS.

Phillip Blanchette and Julia Klein, Motherisk Program, The Hospital for Sick Children, Toronto.

To accurately estimate fetal exposure to constituents of cigarette smoke, we have developed the use of neonatal and maternal hair measures of nicotine and cotinine as biomarkers of fetal exposure.

There is strong correlation between maternal and neonatal levels, and smoking history (no exposure, passive or active smoking) are well reflected in hair.

Because maternal hair grows 1-1.5 cm/ mo, these measures can document smoking history (or abstinence) over time, as well as gestational changes in nicotine metabolism when smoking is stable.
