# Supplementary material for: Meeting materials from the 2003 Annual Meeting of the International Society for the Prevention of Tobacco Induced Diseases
Source: Tob Induc Dis. 2003 Dec 15;1(4):234. doi: 10.1186/1617-9625-1-4-234 (PMC2671532; doi:10.1186/1617-9625-1-4-234)
Supplement: Additional file 1 [file 1617-9625-1-4-234-S1.zip › Abstract 5-An Introduction to Tobacco-Induced Oral Malignancy.pdf]

Abstract 5

An Introduction to Tobacco-Induced Oral Malignancy: Types of Cancer, Etiology and Diagnosis.

Catalena Birek, University of Manitoba, Canada.

Tumors of the head and neck region are broadly classified by site: those of the upper aerodigestive tract (including the oral cavity), the salivary glands and the jaws. The term “head and neck cancer” generally refers to squamous cell carcinoma (SCC) arising in the mucosal lining of the mouth, pharynx and larynx, as this type of cancer represents the majority of malignant tumors found at this anatomical region.

The causal relationship between tobacco use and the development of head and neck cancer is well established.

This introduction to tobacco-induced oral malignancy will comprise discussions on prevalence, etiological co-factors, clinical manifestations (including premalignant lesions of the lips and oral cavity), modern diagnostic procedures and treatment modalities. The need for prevention strategies, and the search for new molecular screening for early detection of oral cancer will be emphasized.
