# Supplementary material for: Meeting materials from the 2003 Annual Meeting of the International Society for the Prevention of Tobacco Induced Diseases
Source: Tob Induc Dis. 2003 Dec 15;1(4):234. doi: 10.1186/1617-9625-1-4-234 (PMC2671532; doi:10.1186/1617-9625-1-4-234)
Supplement: Additional file 1 [file 1617-9625-1-4-234-S1.zip › Abstract 6-Animal models of smokeless tobacco-induced oral malignancies.pdf]

Abstract 6

Animal models of smokeless tobacco-induced oral malignancies

Ernie Lam, University of Alberta, Canada

Peroxynitrite (ONOO $^-$), the reaction product of nitric oxide ($^{\bullet}$NO) and superoxide ($O_2^{\bullet -}$) has been implicated as an important modulator of lipid, protein and DNA damage. Several studies have already demonstrated $O_2^{\bullet -}$ and hydrogen peroxide (H_2O_2) release from tobacco xenobotics, an effect that can be attenuated *in vitro* by recombinant superoxide dismutase (SOD) and catalase. Peroxynitrite formation is favoured in cells when $^{\bullet}$NO and $O_2^{\bullet -}$ are elevated, or when SOD is low, as is the case in tumour cells. Recently, we have demonstrated that tobacco xenobiotic compounds such as smokeless tobacco, nicotine, nitrosonornicotine (NNN) and 4-(methyl-N-nitrosamino)-1-(3-pyridyl)-1-butanone (NNK) have the capacity to release $^{\bullet}$NO in nano- to micromole quantities. We have exposed both immortalized hamster cheek pouch cells (POII) and intact hamster cheek pouch tissues to tobaccoxenobiotics to illustrate the nitrosative effects of ONOO$^-$ in these systems. Lipid peroxidation was quantified in POII cells using an ELISA-based 8-isoprostane assay, and DNA damage was assayed by COMET assay. Because lipid peroxidation and DNA damage are not specific markers of ONOO$^-$ damage, we used immunoflourescence techniques to identify a specific marker for ONOO$^-$: mitrotyrosine. Formation of nitrotyrosine adducts was also identified in hamster cheek pouch tissues chronically exposed to tobacco xenobiotic-derived $^{\bullet}$NO, and that markers of this damage to lipid, protein, and DNA are quantifiable in both cell cultures and intact animals. This work was supported by grants from CIHR and AHFMR.
