# Supplementary material for: Meeting materials from the 2003 Annual Meeting of the International Society for the Prevention of Tobacco Induced Diseases
Source: Tob Induc Dis. 2003 Dec 15;1(4):234. doi: 10.1186/1617-9625-1-4-234 (PMC2671532; doi:10.1186/1617-9625-1-4-234)
Supplement: Additional file 1 [file 1617-9625-1-4-234-S1.zip › Abstract 7-Tobacco-Genetic Interactions and Cancer Induction.pdf]

Abstract 7

Tobacco-Genetic Interactions and Cancer Induction.

Philip Lazarus, Cancer Epidemiology & Prevention Program, H. Lee Moffitt Cancer Center University of South Florida, USA.

Dr. Philip Lazarus' research group focuses primarily on the elucidation of mechanisms involved in the induction and progression of tobacco-related cancers, including molecular epidemiologic studies examining genetic polymorphisms in xenobiotic-metabolizing enzymes as markers for cancer risk. The carcinogenic activity of tobacco carcinogens can often depend on the cellular composition of P450s and various other enzyme systems that are involved in the activation or detoxification of many tobacco carcinogens. The expression and/or activity of these enzymes may be a critical determinant of individual risk for tobacco carcinogen-induced damage. One such enzyme family is the UDP-glucuronosyltransferase family of enzymes, which are responsible for the glucuronidation and detoxification of a variety of carcinogens. Studies in Dr. Lazarus' lab have identified the major enzymes responsible for the glucuronidation of several major tobacco/tobacco smoke carcinogens, including metabolites of benzo(a)pyrene and the nicotine-derived tobacco-specific nitrosamine, 4-(methylnitrosamino)-1-(3-pyridyl)-1-butanone (NNK). Dr. Lazarus' group has identified which of these enzymes are expressed in specific target tissues, and has identified a number of protein-altering genetic polymorphisms that are associated with a number of these enzymes. Case:control studies from Dr. Lazarus' laboratory have demonstrated that polymorphisms present in some of the genes that code for these enzymes may be involved in increased risk for tobacco-related cancers. These polymorphisms have been shown to modulate metabolizing enzyme activity in specific tissues, demonstrating a correlation between metabolizing enzyme genotype and carcinogen-metabolizing phenotype. These studies implicate specific metabolism pathways as important determinants of risk for tobacco-related cancers and that they may be important targets for future chemoprevention strategies.
