# Supplementary material for: Meeting materials from the 2003 Annual Meeting of the International Society for the Prevention of Tobacco Induced Diseases
Source: Tob Induc Dis. 2003 Dec 15;1(4):234. doi: 10.1186/1617-9625-1-4-234 (PMC2671532; doi:10.1186/1617-9625-1-4-234)
Supplement: Additional file 1 [file 1617-9625-1-4-234-S1.zip › Abstract 9-Smokeless tobacco use among adolescent athletes.pdf]

Abstract 9

.

Smokeless tobacco use among adolescent athletes.

Dennis N. Ranalli, School of Dental Medicine, University of Pittsburgh, PA., USA

The adolescent period has been well-documented as the developmental stage in which high risk behaviors are initiated that may affect the general and oral health of the individual throughout the life span. One such behavior is the use of smokeless tobacco products by adolescent athletes. Nicotine addiction from smokeless tobacco may be the result of hero worship for a professional athlete role-model, peer pressure by teammates, the stimulation derived from a nicotine "buzz" or the desire to suppress appetite by participants in weight limit sports.

The purpose of this presentation is to provide information to physicians and dentists regarding the oral manifestations of smokeless tobacco use in adolescent athletes. Included among these conditions will be visible effects on the dentition as well as soft tissue changes to the periodontium and oral mucosa such as gingival recession, alveolar bone loss, leukoplakia and squamous cell carcinoma.

The diagnostic steps in the brush biopsy technique will be presented to aid practitioners in the early detection of suspicious lesions along with histological sections of these soft tissue changes. The devastating consequences of therapeutic interventions and dental rehabilitation will be addressed. Behavioral modification techniques along with pharmacological management cessation strategies will be provided. A video demonstrating a systematic oral cancer screening examination will be shown.

Conclusion: Physicians and dentists must become more proactive in the education of adolescent athletes to the dangers of smokeless tobacco use and to dispel the notion that smokeless tobacco is a safe alternative to cigarette smoking.
