# Supplementary material for: Meeting materials from the 2003 Annual Meeting of the International Society for the Prevention of Tobacco Induced Diseases
Source: Tob Induc Dis. 2003 Dec 15;1(4):234. doi: 10.1186/1617-9625-1-4-234 (PMC2671532; doi:10.1186/1617-9625-1-4-234)
Supplement: Additional file 1 [file 1617-9625-1-4-234-S1.zip › Abstract 10-Electronic Medical Record Tobacco Use Vital Sign.pdf]

Abstract 10

Electronic Medical Record Tobacco Use Vital Sign.

John W. Norris III* and David J. Murphy, UMDNJ, New Jersey, USA.

Objective: Determination of a busy clinical practice's tobacco use prevalence and therapeutic impact utilizing an electronic medical record enabled tobacco vital sign to demonstrate overall practice tobacco prevalence and impact of tobacco prevention/treatment efforts.

Methods: Retrospective cohort study utilizing patient data recorded in an electronic medical record database between July 15, 2001 and January 15, 2003. Patient reported tobacco use status was obtained and recorded for each of 5611 patients during the pre-provider period of their 14206 individual patient visits during the study period with the recorder blinded to past tobacco use status entries recorded for that patient.

Results: An overall current tobacco use prevalence of 26.7% was found during the study period. Tobacco use status was recorded in 97% of visits the total visits. Comparison of aggregated tobacco use status by visit demonstrated a trend toward compliance with tobacco cessation with increased visit utilization. Comparison of initial to final visit tobacco use status demonstrates a consistency rate of 73.5% declaring no change in tobacco status in the 3636 patients with 2 to more visits. A 5.6% net tobacco use decline was seen for the practice.

Conclusions: Self reported tobacco-use-status as a vital sign embedded within the workflow of an electronic medical record enabled practice provided a quantitative tool for determination of tobacco use prevalence and trending over the 18-month study period.
