# Supplementary material for: Meeting materials from the 2003 Annual Meeting of the International Society for the Prevention of Tobacco Induced Diseases
Source: Tob Induc Dis. 2003 Dec 15;1(4):234. doi: 10.1186/1617-9625-1-4-234 (PMC2671532; doi:10.1186/1617-9625-1-4-234)
Supplement: Additional file 1 [file 1617-9625-1-4-234-S1.zip › Abstract 11-Epidemiology of Tobacco Use in Sweden, the Country with Europe's Lowest.pdf]

Abstract 11

Epidemiology of Tobacco Use in Sweden, the Country with Europe's Lowest Level of Tobacco-Related Disease.

Lars M. Ramström*, Institute for Tobacco Studies, Stockholm, Sweden.

Objective: To analyze the development of patterns of different kinds of tobacco use in relation to patterns of tobacco-related disease.

Methods: Combining data from different statistical sources including a recent study on use of cigarettes and smokeless tobacco in a nationwide representative sample of the Swedish population.

Results: During the last 30 years the prevalence of daily smoking among males has decreased by about 25 percentage points (from 40% to 15%), while prevalence of daily use of 'snus' (the special Swedish kind of oral smokeless tobacco) has increased by about 10 percentage points (from 10% to 20%). At the same time the prevalence of daily smoking among women has decreased by about 13 percentage points (from 33% to 20%), while the prevalence of female daily use of snus is still low, about 2%. Smoking cessation rates are equal for women and for men without use of snus, while men with a history of snus use have substantially higher cessation rates, as explained by the finding that a majority of successful male quitters report use of snus as a cessation aid. In males the decrease of smoking has been followed by a decrease in all smoking-related diseases at the same time as the increased use of snus has not been associated with an increase of oral cancer.

Conclusions: Tobacco-related disease in Sweden appears to be primarily related to smoking rather than to total tobacco use including the Swedish smokeless tobacco.
