# Supplementary material for: Meeting materials from the 2003 Annual Meeting of the International Society for the Prevention of Tobacco Induced Diseases
Source: Tob Induc Dis. 2003 Dec 15;1(4):234. doi: 10.1186/1617-9625-1-4-234 (PMC2671532; doi:10.1186/1617-9625-1-4-234)
Supplement: Additional file 1 [file 1617-9625-1-4-234-S1.zip › Abstract 12-The Effect on FEV1 of Intermittent Smoking vs Sustained Reduction Among.pdf]

## Abstract 12

### *The Effect on $FEV_1$ of Intermittent Smoking vs Sustained Reduction Among Smokers Preparing to Quit in the Lung Health Study*

Robert Murray*, University of Manitoba, Canada, Paula G. Lindgren and John E. Connett, University of Minnesota, USA.

The Lung Health Study enrolled 5,887 smokers who had early evidence of airways obstruction in a randomised clinical trial. The original purpose was to demonstrate the effectiveness of smoking intervention versus usual care on the progression of chronic obstructive pulmonary disease. The primary outcome variable was forced expiratory volume in 1 second ($FEV_1$). Participants were followed over 5 years. Those who quit smoking in the first year of the study and stayed quit lost an average of 0.33%/year in $FEV_1$ of predicted. Those who continued to smoke throughout the study lost 1.18%/year, and those who made multiple attempts to quit smoking lost 0.58%/year. Participants who chose smoking reduction instead of quitting were also followed. Those continuing smokers who did not reduce lost an average of 0.98%/year in $FEV_1$ of predicted. Those who sustained a reduction of 25% or more of their baseline smoking level lost an average of 1.08%/year. Those who sustained a reduction of 50% or more lost an average of 1.26%/year. Quitting smoking for an interval followed by relapse to smoking provided a measurable and lasting benefit in comparison to continuous smoking. Reduction in smoking, on the other hand, was associated with greater lung function loss than was continuous smoking in these data. Multiple attempts to quit confer a benefit in lung function that smoking reduction does not.
