# Supplementary material for: Meeting materials from the 2003 Annual Meeting of the International Society for the Prevention of Tobacco Induced Diseases
Source: Tob Induc Dis. 2003 Dec 15;1(4):234. doi: 10.1186/1617-9625-1-4-234 (PMC2671532; doi:10.1186/1617-9625-1-4-234)
Supplement: Additional file 1 [file 1617-9625-1-4-234-S1.zip › Abstract 14-Mechanisms underlying susceptibility to periodontal and vascular diseases.pdf]

Abstract 14

Mechanisms underlying susceptibility to periodontal and vascular diseases in tobacco smokers

Denis F. Kinane*, University of Louisville School of Dentistry, Louisville, KY, USA.

Numerous investigations of the relationship between smoking and periodontal disease have been performed over the last fifteen years and there now exists a substantial body of literature upon which this current review is based. From both cross-sectional and longitudinal studies, there appears to be strong epidemiological evidence that smoking confers a considerably increased risk of periodontal disease and vascular problems such as atheroma. The association between periodontal disease and smoking is further supported by the data emanating from patients who stop smoking. These patients have levels of risk similar to that of non-smokers. Numerous studies of the potential mechanisms whereby smoking tobacco may predispose to periodontal disease and cardiovascular disease have been conducted and it appears that smoking may affect the vasculature, the humoral immune system, the cellular immune and inflammatory system and have effects throughout the cytokine and adhesion molecule network as well as many potentially confounding aspects whereby smoking in itself may indicate poor health beliefs and practices which would render the individual susceptible to many diseases. The aim of this review is to consider the evidence for the association between smoking and periodontal and atheromatous diseases and to highlight the biological mechanisms whereby smoking may affect the periodontium and the endothelium.
