# Supplementary material for: Meeting materials from the 2003 Annual Meeting of the International Society for the Prevention of Tobacco Induced Diseases
Source: Tob Induc Dis. 2003 Dec 15;1(4):234. doi: 10.1186/1617-9625-1-4-234 (PMC2671532; doi:10.1186/1617-9625-1-4-234)
Supplement: Additional file 1 [file 1617-9625-1-4-234-S1.zip › Abstract 15-Periodontitis, Smoking, and Adverse Pregnancy Outcome.pdf]

Abstract 15

Periodontitis, Smoking, and Adverse Pregnancy Outcome

Suzanne Moore*, Guy's & St. Thomas' Hospital Trust, London, UK

Periodontal disease (or "gum disease") is a common cause of dental morbidity. This inflammatory disease is caused primarily by host response to dental plaque but has several other aetiological variables such as smoking, poor oral hygiene and genetic factors. It has been postulated that the presence of severe periodontal disease in pregnancy is associated with adverse pregnancy outcome, such as premature birth or low birth weight birth.

The results of a prospective study, containing around four thousand subjects, will be presented. Demographic and obstetric data will be discussed alongside smoking status and periodontal health variables. Subjects were taken from a population of pregnant women from Guy's and St Thomas' Hospital Trust, London, UK.

The research involves the investigation of putative mechanisms for adverse pregnancy outcome and attempts to identify risk factors, such as smoking. The work is pertinent to both dental and obstetric research.
