# Supplementary material for: Meeting materials from the 2003 Annual Meeting of the International Society for the Prevention of Tobacco Induced Diseases
Source: Tob Induc Dis. 2003 Dec 15;1(4):234. doi: 10.1186/1617-9625-1-4-234 (PMC2671532; doi:10.1186/1617-9625-1-4-234)
Supplement: Additional file 1 [file 1617-9625-1-4-234-S1.zip › Abstract 16-The Cellular Effects of Tobacco Smoke on the Human Vasculature.pdf]

Abstract 16

The Cellular Effects of Tobacco Smoke on the Human Vasculature

Toru Kato*, Tokyo Medical and Dental University, Japan

Human vasculature is lined with a thin layer of cells named endothelium. Endothelium helps the blood vessels dilate physiologically. Endothelial dysfunction is an early feature of atherogenesis. Endothelial injury predisposes to less handling the change of the blood flow, thrombosis, leucocyte adhesion, and proliferation of smooth muscle cells in the arterial wall.

Even after short-term exposure to tobacco smoke, nonsmokers are prone to be attacked by an excess of free radicals inducing oxidative stress. Tobacco smoke rapidly breaks down the serum antioxidant defense, leading to endothelial dysfunction. That is, we can say even short-term exposure to tobacco smoke is dangerous for nonsmokers.

Recently, communities throughout the world including Japan are beginning to clear the air by making smoke-free workplaces and public places such as restaurant, hospitals, and stations. Tobacco smoke has been identified as an important risk factor for cardiovascular disease, however, it is avoidable. Because nonsmokers are more sensitive and likely to suffer from tobacco smoke than smokers, nonsmokers should be protected from even short-term exposure to tobacco smoke.
