# Supplementary material for: Meeting materials from the 2003 Annual Meeting of the International Society for the Prevention of Tobacco Induced Diseases
Source: Tob Induc Dis. 2003 Dec 15;1(4):234. doi: 10.1186/1617-9625-1-4-234 (PMC2671532; doi:10.1186/1617-9625-1-4-234)
Supplement: Additional file 1 [file 1617-9625-1-4-234-S1.zip › Abstract 17-Tobacco-Genetic Interactions in Vascular Disease.pdf]

## Abstract 17

### ***Tobacco-Genetic Interactions in Vascular Disease.***

*Xing Li Wang*, Jian Wang. Division of Cardiothoracic Surgery, and Michael E. DeBakey Department of Surgery, Baylor College of Medicine, Houston, Texas.*

Despite the decades of preventive efforts, cardiovascular diseases remain the No 1 killer in USA and most industrialized countries. With intensive research efforts, we have acquired considerable knowledge with regard to both environmental risk factors, such as high-fat diet, and genetic risk factors, such as LDL receptor mutation. However, our understanding of the specific relationships between the gene-environment interaction and vascular disease has only started in recent years. Tobacco smoking as an addictive habit is one of the most potent and prevalent environmental health risks humans are exposed to and responsible for more than 1000 death each day in USA. The pathogenic effect is not only present in active smokers but also innocent passive smokers. While the cigarette-induced harm to human health is indiscriminant and severe, the degree of damage also varies from individual to individual. This inter-subject variability in cigarette-induced pathologies is partly mediated by genetic variants of genes that may participate in detoxification process, e.g. cytochrome P450 (CYP), cellular susceptibility to toxins, such as p53, or disease development. Genetically determined susceptibility to cigarette smoking may provide an ideal model for investigating tobacco-gene interaction in pathogenesis. Through population studies, we have learnt that certain CYP1A1 variants, such as *Msp*I polymorphism, may render the carriers more susceptible to cigarette-induced lung cancer or severe coronary atherosclerosis. The endothelial nitric oxide synthase intron 4 rare allele homozygotes are more likely to have myocardial infarction if they also smoke. *In vitro* experiments have further demonstrated that cigarettes may specifically regulate eNOS expression in a genotype-dependent fashion. Particular p53 genotype carriers appear to be more susceptible to smoking induced vascular diseases. While we still know little about genetic basis and molecular pathways for cigarette-induced pathological changes, understanding these mechanisms will be of great value in designing strategies to encourage smoking cessation, and to implement more effective measures in prevention and treatment of cigarette-induced diseases.
