# Supplementary material for: Meeting materials from the 2003 Annual Meeting of the International Society for the Prevention of Tobacco Induced Diseases
Source: Tob Induc Dis. 2003 Dec 15;1(4):234. doi: 10.1186/1617-9625-1-4-234 (PMC2671532; doi:10.1186/1617-9625-1-4-234)
Supplement: Additional file 1 [file 1617-9625-1-4-234-S1.zip › Abstract 18-Smoking Cessation at the Municipal Level in Japan.pdf]

Abstract 18

Smoking Cessation at the Municipal Level in Japan.

Kazunari Satomura*, Toshitaka Nakahara, Kazuaki Miyagishima, Fumiko Matsuda, Asuka Komatsu, Megumi Noami and Takehiko Sakurami.

One of the targets of Healthy Japan 21 is decreasing smoking rates. To evaluate efforts of municipalities, a survey of their relationship between municipalities and organizations was performed.

Subjects and Methods: A questionnaire that was asking their relationship in 2002 was sent to all municipalities in Japan.

Results: 1410 out of 3244 municipalities replied to the questionnaire. 11.5% of municipalities had information of smoking cessation programs in public hospitals, 7.3% of them had that in private hospitals, 23.3% of them had that in other municipalities, 33.4% of them had that in public health centers, 14.5% of them had that in schools and 1.5% of them had that in non-profit or non-government organization. 2.7% of municipalities were requested cooperation in smoking cessation programs from public hospitals, 2.2% of them from private hospitals, 6.7% of them from other municipalities, 12.7% of them from public health centers, 14.8% of them from schools and 1.3% of them from non-profit or non-government organization. 10.5 of municipalities gave information of stop smoking to public hospitals, 11.7% of them to private hospitals, 16.9% of them to other municipalities, 16.3% of them to public health centers, 16.2% of them to schools and 3.2% of them from non-profit or non-government organization.

Discussion: However municipalities should be chief promoters in Healthy Japan 21, these results show that they played a little role in smoking cessation programs. To reduce smoking rates, cooperation between municipalities and other organizations are necessary.

Conclusion: Municipalities need new strategies for cooperation in quit smoking.
