# Supplementary material for: Meeting materials from the 2003 Annual Meeting of the International Society for the Prevention of Tobacco Induced Diseases
Source: Tob Induc Dis. 2003 Dec 15;1(4):234. doi: 10.1186/1617-9625-1-4-234 (PMC2671532; doi:10.1186/1617-9625-1-4-234)
Supplement: Additional file 1 [file 1617-9625-1-4-234-S1.zip › Abstract 19-Evaluation of the Kick Butt Smoking Cessation Program at the Wellness.pdf]

Abstract 19

Evaluation of the Kick Butt Smoking Cessation Program at the Wellness Institute, Manitoba, Canada.

Dr. Kevin Saunders*, Sherry Mooney, Erica White*, Nicole Dunn. The Wellness Institute, Manitoba, Canada.

Background: The Wellness Institute at Seven Oaks General Hospital opened as the first Medical Fitness Facility in Canada in 1996. In that year 1861 Manitobans died of tobacco-related deaths. In 1998, staff at the Wellness Institute recognized that the program currently offered had failed to assist even one Manitoban to quit tobacco use. A literature review revealed that the Mayo Clinic reported the highest success rates of 43-45% using an addiction treatment model. In 1999 the Wellness Institute developed the *Kick Butt* program, modelled after the successful Mayo Clinic approach.

Program Description: The Kick Butt program addresses tobacco use as an addiction. The program focuses on individuals with flexible intakes, recognizes and treats both the addiction and the behaviour aspects of tobacco use. The program consists of four components: 1) Pharmacology; 2) Behaviour counselling; 3) Replacement activities; and 4) Relapse prevention and aftercare. Program services include a medical assessment, four behaviour therapy sessions and a lifestyle/fitness assessment and facility access over a three month period.

Results: In its pilot phase, Kick Butt had a 43% success rate one year post quit. Subsequent follow up from 200-2002 reveals a quit rate of 60%.

Conclusion: Kick Butt has a higher success rate than self-help programs, on-line programs, and other group and individual programs reviewed. It is the most successful program offered to date in Winnipeg.
