# Supplementary material for: Meeting materials from the 2003 Annual Meeting of the International Society for the Prevention of Tobacco Induced Diseases
Source: Tob Induc Dis. 2003 Dec 15;1(4):234. doi: 10.1186/1617-9625-1-4-234 (PMC2671532; doi:10.1186/1617-9625-1-4-234)
Supplement: Additional file 1 [file 1617-9625-1-4-234-S1.zip › Abstract 20-Smoking Cessation Using the Internet.pdf]

Abstract 20

Smoking Cessation Using the Internet.

Yuko Takahashi*, Nara Women's University, Nara; Akiko Higashiyama, Kansai Fukushi University, Akoh; and Hideshi Miura, NEC Nexsolution Co., Ltd., Tokyo, Japan.

Background; Since 1997 we have been running the Internet mediated health support for quitting aiming for to have smokers quit for the rest of their lives; the Quit Smoking Marathon.1683 smokers participated.
Objectives; The Internet mediated program for smokers contains a brief motivational intervention, personalized and real-timed feedback, and motivational enhancement. We aim to compare the rate of abstinence in participants who took in part of the year email program and not.
Methods; One year smoking rate and other questionnaire were checked by three Methods, email self-report, telephone call to their families or co-workers and mail.
Results; After one year from the registration, we followed up 194 participants who registered the program in 2000 and checked their smoking status. We could not follow 37 mainly because of the participants transference with removal. The 1 year quitting rate is 63.6% of the participants who had registered and 75.8% we followed by usual method.
We have found significant difference in quit rates at one year out between the participants who took in part in participants of the year email program and not (89.4% .vs. 56.1%). No significant differences were found in sex or in nicotine dependency.
Conclusion; The Internet mediated health supporting program aiming for to have smokers maintain quitting may be the effective program in quitting.
