# Supplementary material for: Meeting materials from the 2003 Annual Meeting of the International Society for the Prevention of Tobacco Induced Diseases
Source: Tob Induc Dis. 2003 Dec 15;1(4):234. doi: 10.1186/1617-9625-1-4-234 (PMC2671532; doi:10.1186/1617-9625-1-4-234)
Supplement: Additional file 1 [file 1617-9625-1-4-234-S1.zip › Abstract 21-Effects of Complete and Partial Smoking Bans on the Respiratory Health of.pdf]

Abstract 21

Effects of Complete and Partial Smoking Bans on the Respiratory Health of Hospitality Workers in Brandon and Winnipeg, Manitoba.

Mark Taylor*, Robert Murray, Morley Lertzman, Kristina Hunter, Nykola Dubenski*, Tricia Kutnikoff. Manitoba Medical Association, Health Sciences Center and University of Manitoba, Canada.

Exposure to environmental tobacco smoke (ETS) is a critical public health issue. ETS exposure is associated with a variety of adverse health impacts, including carcinogenicity and cardiovascular, developmental, reproductive, and childhood respiratory effects. Hospitality workers are among the workers at greatest risk of exposure to ETS in the workplace. For example, in a typical eight-hour shift, a bartender can inhale the ETS equivalent to smoking 36 cigarettes, depending on ventilation rates.

Objectives:

- To measure indicators of ETS exposure in the workplace on non-smoking hospitality workers.
- To determine if non-smoking hospitality workers in smoking venues have greater reported health symptoms and greater ETS exposure indicators than those in non-smoking venues.
- To determine if non-smoking hospitality workers in venues with a full smoking ban have fewer reported health symptoms and lower ETS exposure indicators than those in non-smoking venues.

Methods: Exhaled carbon monoxide levels and peak flow were measured before and after a work shift to assess the effects of ETS exposure over the shift. Interviews were conducted with participants in order to assess respiratory symptoms, sensory irritation symptoms, personal smoking, and other ETS exposure.

Results and conclusions: Those tested were all nonsmoking staff, working in smoking or nonsmoking environments, the latter with a recent nonsmoking bylaw in effect. Total reported symptoms in the smoking venues were significantly greater (mean 3.00) than those reported in non-smoking venues (mean 1.23), as predicted. Increased levels of exhaled carbon monoxide over the work shift were significantly greater in the smoking venues compared to bylaw venues, but the difference between smoking and non-smoking environments was only marginally significant.
