# Supplementary material for: Meeting materials from the 2003 Annual Meeting of the International Society for the Prevention of Tobacco Induced Diseases
Source: Tob Induc Dis. 2003 Dec 15;1(4):234. doi: 10.1186/1617-9625-1-4-234 (PMC2671532; doi:10.1186/1617-9625-1-4-234)
Supplement: Additional file 1 [file 1617-9625-1-4-234-S1.zip › Abstract 22-Circulating Matrix Metalloproteinase Profiles.pdf]

Abstract 22

Circulating Matrix Metalloproteinase Profiles in Tobacco Smokers and Non-Smokers.

Jennifer T Borden*[1] and David A Scott[1,2]. Oral Biology[1] and Dental Diagnostics & surgical Sciences[2], University of Manitoba, Canada.

Background: Degradation of collagen, and other connective tissue macromolecules, by matrix metalloproteinases (MMPs) is considered to play an important role in the initiation and progression of various diseases including periodontitis, vascular diseases, pulmonary emphysema, and certain cancers. Yet the influence of tobacco smoke on MMP release is, essentially, unstudied.

Objectives: To compare the species, form, and quantity of MMP family members in the circulation of smokers and non-smokers.

Materials and Methods: The self-reported smoking status of 19 smokers and 20 age- and gender-matched non-smokers was validated by expired-air carbon monoxide measurement and by serum cotinine analysis. MMP profiling was accomplished by zymography, using gelatin, collagen (types I and IV), and casein as substrates. MMP activity levels were determined by densitometry. MMP-inhibition (EDTA), serine protease inhibition (PMSF), Western blotting, and ELISA were used to confirm MMP identities.

Results: Active and pro-MMP-2, -MMP-3, -MMP-8, and -MMP-9 were detected in serum of both smokers and non-smokers. Total MMP-9 concentration was increased in the serum of smokers, compared to non-smokers ($p < 0.05$). MMP-9-NGAL (a neutrophil specific molecule) complexes were also elevated in smokers, suggesting the neutrophil to be the cellular source of this increased MMP-9 burden.

Conclusions: Systemic concentrations of total, neutrophil-derived MMP-9 appear to be significantly elevated in tobacco smokers. MMP-9 release from neutrophils may be a common pathological mechanism underlying several tobacco-induced diseases. Further studies to examine endogenous MMP inhibitors and to confirm these initial data in an *in vitro* model are currently underway.
