# Supplementary material for: Meeting materials from the 2003 Annual Meeting of the International Society for the Prevention of Tobacco Induced Diseases
Source: Tob Induc Dis. 2003 Dec 15;1(4):234. doi: 10.1186/1617-9625-1-4-234 (PMC2671532; doi:10.1186/1617-9625-1-4-234)
Supplement: Additional file 1 [file 1617-9625-1-4-234-S1.zip › Abstract 23-Delayed Oral Wound Healing in Tobacco Smokers.pdf]

Abstract 23

Delayed Oral Wound Healing in Tobacco Smokers

Babak Rashidkhani*, South Tehran Health Center, Tehran University of Medical Sciences, Tehran, Iran.

Background and Aim: The purpose of this study was to determine the effects of cigarette smoking on the healing process of oral wounds caused by the extraction of posterior teeth.
Materials and Methods: Two groups were chosen for the study. The test group comprised 47 smokers and the control group comprised 52 non-smokers. In both groups the age ranged from 30 to 50. In both groups there were no systemic diseases and were candidates for extraction of posterior teeth (4,5,6,7 and 8). About one week to 10 days after the operation, the both groups were re-in view of recovery of their wounds. In study of the recovery, variables such as coming close of the edges of the wound, filling of the cavity resulting from tooth extraction (socket), existence or lack of inflammation in the area were studied.
Results: The findings indicate a significant relationship between cigarette smoking and wound recovery. The possibility of delay in recovery of wounds in smokers is 3.19 times more than non-smokers. Delayed wound healing was observed in 55.3% of the smokers, while the corresponding figure for non-smokers is just 17.3%. Also the findings indicate no significant relationship between gender-age factors and wound healing in the age range studied.
Conclusions: The results of this study confirm that there is a correlation between smoking and delayed oral wound healing.
