# Supplementary material for: Meeting materials from the 2003 Annual Meeting of the International Society for the Prevention of Tobacco Induced Diseases
Source: Tob Induc Dis. 2003 Dec 15;1(4):234. doi: 10.1186/1617-9625-1-4-234 (PMC2671532; doi:10.1186/1617-9625-1-4-234)
Supplement: Additional file 1 [file 1617-9625-1-4-234-S1.zip › Abstract 24-Smoking, Nicotine, and Pancreatic.pdf]

Abstract 24

Smoking, Nicotine, and Pancreatic Dysfunction.

Parimal Chowdhury* and Kodethoor B. Udupa. University of Arkansas, Little Rock, AR, USA.

Many epidemiological studies linked smoking as an important factor for induction of chronic pancreatitis. The mechanism, however, is yet unknown. Since nicotine, a component of cigarette smoke is considered a risk factor for the pathogenesis of many diseases including pancreas we wondered whether or not nicotine directly affects pancreatic function. Earlier studies conducted in rats with nicotine exposure revealed morphological changes in the exocrine pancreas. The goal of the current study was to examine whether nicotine exposure induces the functional abnormality in an in vitro stable primary cell culture with isolated pancreatic acini.

Methods: Fasted, anesthetized rats were sacrificed by decapitation. Pancreas was quickly removed and processed for isolation of pancreatic acini. Isolated acini were incubated in HR buffer with 0 to 30 mM doses of nicotine at 37^{0}C for 30 min. Incubated acini were centrifuged, washed with HR buffer, dispersed and stimulated with a maximal stimulating dose of carbachol (10^{-5} M). The nicotine dose response curve revealed a 50% inhibition of amylase release at a nicotine dose of 4.79 mM. Five subsequent experiments were conducted in dispersed pancreatic acini that were divided into four groups each for control and nicotine (5 mM) and cultured for 30 min, 36 hrs, 72 hrs and 108 hrs respectively at 37^{0}C in 5% CO_2 and 95% O_2 atmosphere in Waymouth's MB752/1 medium. At the end of each culture period, cells were washed, resuspended in fresh HR and then incubated for 30 min at 37^{0}C with or without carbachol. Cells were counted before, during and at the end of the culture periods. Basal release of amylase was measured in the medium. DNA, protein and amylase release for each group were measured in response to carbachol.

Results: There was no difference in cell numbers and protein concentration between control and nicotine exposed acini cultures for periods of 72 hrs. Thereafte,r cell numbers and protein concentrations were decreased in both groups. DNA change was stable for 36 hrs and decreased dramatically for the rest of the culture period in both groups. Amylase release in the medium was not different at 0 h between the two groups but was consistently higher for nicotine group for 36 to 108 hr. periods. Cell function measured in response to carbachol was significantly lower for nicotine group for all time periods of culture, $p<0.05$.

Conclusions: The data suggest that nicotine modulates acinar cell function in vitro very early with no effects on DNA and protein.
