# Supplementary material for: Meeting materials from the 2003 Annual Meeting of the International Society for the Prevention of Tobacco Induced Diseases
Source: Tob Induc Dis. 2003 Dec 15;1(4):234. doi: 10.1186/1617-9625-1-4-234 (PMC2671532; doi:10.1186/1617-9625-1-4-234)
Supplement: Additional file 1 [file 1617-9625-1-4-234-S1.zip › Abstract 25-Genetic susceptibility and smoking intensity.pdf]

Abstract 25

Genetic susceptibility and smoking intensity

Thanos Zavras*, Harvard University, USA.

The worldwide prevalence of tobacco use is widespread, resulting in nearly 4.5 million deaths very year.

As international trends indicate, tobacco consumption is decreasing in industrial countries, but increasing in the developing world; future consequences are not limited to increased morbidity and mortality, but also include a serious social and economic burden.

Several studies have documented a strong hereditary component to tobacco use, including family, twin and association studies. The hereditary component seems to be present in both initiation and addiction.

Our presentation is composed of two sections: it first reviews the magnitude of association for a number of specific psychoactive or metabolic genes that may be involved in tobacco use. The second part describes own results from an epidemiological case-control investigation of drd2 polymorphisms and smoking. The main finding was that a dopaminergic haplotype, dopamine receptor DRD2-A2B2, was found associated with intensity of smoking ($p<0.05$).
