# Supplementary material for: Meeting materials from the 2003 Annual Meeting of the International Society for the Prevention of Tobacco Induced Diseases
Source: Tob Induc Dis. 2003 Dec 15;1(4):234. doi: 10.1186/1617-9625-1-4-234 (PMC2671532; doi:10.1186/1617-9625-1-4-234)
Supplement: Additional file 1 [file 1617-9625-1-4-234-S1.zip › Abstract 26-Smoking adversely affects arterial endothelial function in apparently healthy.pdf]

Abstract 26

Smoking adversely affects arterial endothelial function in apparently healthy Chinese subjects

G Neil Thomas[1*], Mui Qiao[2], Ping Chook[2], Xin S Huang[3], Jiang Zheng Feng[4], Peter YK Poon[2], Guo G Wang[5], Shu W Chan[6], Hok C Leong[7], David S Celermajer[8], Kam Sang Woo[2].

[1]Department of Community Medicine, The University of Hong Kong, Pokfulam, Hong Kong; [2]Department of Medicine and Therapeutics, The Chinese University of Hong Kong, The Prince of Wales Hospital, Shatin, Hong Kong, [3]Guangdong Provincial Hospital and [4]Guangdong Provincial Cardiovascular Institute, Guangzhou, [5]Cardiovascular Institute and Fuwai Heart Hospital, Beijing, China, [6]The Chinese Hospital, San Francisco, USA, [7]Kiang Wu Hospital, Macau, [8]Departments of Medicine and Cardiology, Royal Prince Alfred Hospital, University of Sydney, Sydney, Australia.

Objective: Endothelial vasodilatatory dysfunction (FMD) and carotid intima-media thickening (IMT) are associated with vascular dysfunction and endothelial damage, which are useful surrogate markers of morbidity and mortality from cardiovascular disease. We report the relationship between brachial artery endothelial function, carotid IMT and smoking.

Methods: FMD, carotid IMT were measured non-invasively by high resolution ultrasound B mode imaging in 533 apparently healthy southern Chinese subjects (22.9% smokers) recruited from greater China and the USA.

Results: When the groups were compared, the smokers had significantly worse FMD (8.2±2.5 vs. 7.0±2.3%, p<0.001) and endothelium-independent vasodilatation (GTN, 18.5±3.3 vs. 17.6±3.0%, p=0.004) and IMT (0.59±0.12 vs. 0.63±0.13 mm, p=0.001). However, there were considerable gender differences in the prevalence of smokers, with 91.0% of the smokers being male. As male gender was associated with adverse vascular function, even in the non-smokers, we assessed the relationship with smoking in the male group. Both endothelium-dependent (8.0±2.5 vs 6.9±2.2%, p<0.001) and independent (18.2±3.7 vs 17.3±3.5%, p=0.047) vasodilatation were significantly lower in the smokers (n=129) than non-smokers (n=174), even though age and cholesterol levels were lower in the smoking group. The lower FMD:GTN in the smokers (0.41±0.12 vs 0.45±0.13, p=0.005) suggests that smoking affected FMD to a greater extent than the GTN.

Conclusion: In these apparently healthy Chinese subjects smoking was adversely associated with endothelial function.
